# Effects of infection and disease with *Mycobacterium tuberculosis* on serum antibody to glucan and arabinomannan: two surface polysaccharides of this pathogen

**DOI:** 10.1186/1471-2334-13-276

**Published:** 2013-06-19

**Authors:** Wendy A Keitel, ZhongDong Dai, Robert W Awe, Robert L Atmar, Sheldon Morris, Rachel Schneerson, John B Robbins

**Affiliations:** 1 Departments of Molecular Virology & Microbiology and Medicine, Baylor College of Medicine, Houston, TX, USA; 2 Program on Developmental and Molecular Immunity, Eunice Schriver Kennedy Institute of Child Health and Human Development, NIH, Bethesda, MD 20852, USA; 3 Ben Taub General Hospital, Houston, TX, USA; 4 CBER, Food and Drug Administration, Houston, TX, USA

**Keywords:** *Mycobacterium tuberculosis*, Capsular polysaccharides, Immune responses

## Abstract

**Background:**

The role of the surface capsular polysaccharides (CPs) of *Mycobacterium tuberculosis* (Mtb) in the pathogenesis of infection and disease, as well their potential for use as diagnostic reagents and vaccine antigens, are unknown.

**Methods:**

Serum antibody to two CPs of Mtb, arabinomannan (AM) and glucan (Glu), were studied in samples from 52 18–74 year-old HIV-seronegative, immunocompetent individuals in Houston Texas. The effects of Mtb exposure, infection and disease upon the levels of antibodies to these CPs were assessed. Subjects were grouped according to the standard international classification.

**Results:**

IgA anti-Glu levels were significantly higher in the active and treated TB compared to a group that was PPD-negative without TB exposure history (p<0.05). Antibodies against AM demonstrated a similar pattern, with the exception that IgG anti-AM was higher in groups who had active TB or previously documented active TB, and IgA anti-AM was higher in subjects with previously documented active TB compared to the level in an unexposed, PPD-negative group (p<0.05). Serum IgG anti-Glu levels were higher in subjects with active TB or previously documented active TB than in the unexposed PPD-negative group, but the differences were not significant.

**Conclusions:**

These data suggest that the evaluation of antibody responses to the CP of Mtb may have utility for TB serodiagnosis, and that vaccines designed to induce humoral responses to TB CPs should be tested for their capacity to evoke anti-tuberculosis protective immunity.

## Background

Despite the availability of chemotherapeutic agents and a vaccine (BCG), Mtb remains the number one cause of death due to bacterial infectious diseases worldwide. In 2011, 8.7 million people developed clinical disease and 1.4 million died from TB [1]. Control of TB has been hampered by the absence of sensitive diagnostic means, the spread of HIV infection, and the emergence of multidrug resistant Mtb. The only licensed vaccine against tuberculosis (BCG) is effective in preventing systemic TB in children, but its efficacy against pulmonary TB in adults is controversial [2]. Improved vaccines and diagnostic assays are needed.

Mtb is an encapsulated organism and its capsule is composed of lipid derivatives of arabinomann (AM) and glucan (Glu) [3-6]. The role of these CPs in pathogenesis of and immunity to Mtb is unknown; however, CPs of other important bacteria such as *Haemophilus influenzae* type b, *Streptococcus pneumoniae, Neisseria meningitidis* and *Salmonella typhi* have been shown to inhibit complement-mediated and phagocytic actions, thereby preventing initial control of infection [7,8]. Antibody to these CPs promote clearance of the organisms. Because the CPs of these bacteria are used for diagnosis and prevention of diseases caused by these pathogens, we evaluated the Mtb CPs as potential diagnostic reagents or vaccines for TB. This pilot study assessed antibody responses to the two CPs of Mtb among immunocompetent subjects who were stratified according to their history of infection with and/or disease caused by Mtb.

## Methods

### Subjects

Male and female subjects ≥ 18 years old (Table 1) were recruited from the Texas Medical Center and from the Harris County Hospital District in Houston, TX between March 1999 and October of 1999. Informed consent was obtained from each participant in accordance with protocols approved by the Institutional Review Board for Human Subject Research for Baylor College of Medicine & Affiliated Hospitals. Review of history of exposure to or infection with Mtb, current medications, and potential immunosuppressive conditions was conducted by clinicians with expertise in pulmonary medicine (RWA) or infectious diseases (WAK). Medical records were reviewed to document diagnoses, treatment and tuberculin skin testing results, as appropriate. All patients with active TB were tested for antibodies to HIV-1: those with no history of active TB were required to have a negative HIV-1 serum antibody assay within a year of blood collection. Subjects who had evidence of HIV-1 infection, immunosuppression or a history of BCG vaccination were excluded.

**Table 1:** Characterization of enrolled subjects

**TB Class**	**Number**	**Gender**	**Mean age in**	**Race/Ethnicity**
		**(F:M)**	Years (SD)*	**(C:AA:H:A)**
0	11	6:5	44.9 (10)	4:4:3:0
1	11	7:4	37.6 (11.2)	3:5:3:0
2	11	6:5	43.6 (10)	4:4:2:1
3	10	4:6	45.9 (14.3)	3:3:4:0
4	9	1:8	43.0 (4.8)	1:6:1:1

Abbreviations: *F* Female, *M* Male, *C* Caucasian, *AA* African-American, *H* Hispanic, *A* Asian.

* p=NS between groups; ANOVA.

### Clinical procedures

Enrolled subjects provided a 20 mL blood sample collected from an arm vein. In addition, one subject with active TB underwent plasmapheresis for collection of plasma for assay standardization. Up to 11 subjects were enrolled into each of the following groups corresponding to the standard international classification of TB [9]:

Group 0 No history/evidence of TB or recent exposure to TB and negative PPD (PPD-negative);

Group I Exposed to TB but no evidence of infection (contact of a case, or exposed);

Group 2 TB infection (positive PPD) but no disease (i.e., latent TB);

Group 3 Active TB;

Group 4 History of active TB with no current disease (previously documented active TB).

### Polysaccharides

The two polysaccharides were purified from a 70% ethanol precipitate of a liquid culture of Mtb strain MT29248. The precipitates were suspended in 0.02 M potassium phosphate, pH 7.4, stirred 2 hours, spun down and the supernatant passed through a DEAE column equilibrated in the same buffer. The non-retarded fraction was concentrated, passed through a CL-4B Sepharose column and the major peak, composed of Glu, freeze-dried. The later eluant fractions were dialyzed against H_2_O, freeze dried and passed through a CL-6B Sepharose column. The single peak in this eluate, composed of AM, was dialyzed and freeze-dried. Both CPs contained <1% protein and nucleic acids [10].

### ELISA

Serum anti-Glu or anti-AM levels were measured by ELISA during year 2000 [11]. Nunc plates (PGC, Frederick, MD) were coated with 100 μL of 10 μg/mL Glu or AM in PBS. Mouse monoclonal anti-human IgG, IgM, or IgA antibodies (IgG HP6043, IgM HP6084, IgA HP6107; Centers for Disease Control and Prevention) were used. Alkaline phosphatase-labeled polyclonal rat-anti mouse was the 2^nd^ antibody (Jackson Immuno Research Lab, Inc). A patient’s plasma obtained by plasmapheresis was used as the standard for the 3 isotypes The isotype-specific concentrations of anti GLU and anti AM in that plasma were assigned by ELISA in comparison to purified IgG, IgM and IgA of known concentrations, as described [12].

### Statistical analyses

Unexposed, PPD-negative subjects (group 0) were considered the reference control group for statistical evaluations of serum antibody levels against the two polysaccharides. Data were not normally distributed, so non-parametric statistical analyses were performed. Median antibody levels between groups were compared using a Kruskall-Wallis (KW) test, and when significant differences were identified, post- hoc comparisons of other groups to group 0 were performed using Dunn’s post hoc test corrected for multiple comparisons. P values ≤ 0.05 were considered significant.

## Results

### Clinical

Fifty-two eligible subjects were enrolled (Table 1). The mean age, sex and race/ethnicity distributions were similar among the groups, with the exception of the group with a history of treated TB, who were mostly African-American males. Among 22 subjects who had no history of TB exposure or infection (groups 0 and 1), 21 had a negative PPD documented within 1 calendar year of enrollment, as most of these subjects were employees of institutions where annual skin testing is required. Among subjects with latent TB (PPD+), the date of the positive PPD ranged from 1959 (retested positive subsequent to sample donation) to 1998, with a mean of 8 years since having a positive test (range=1-19 years). Most of these subjects worked in healthcare settings. Among patients with active TB, the most common diagnosis was pulmonary TB (9/10); the remaining subject had a mediastinal mass with necrotizing granulomas. The mean time between diagnosis of TB and collection of the blood sample was 3 months (range <0.5 to 12 months). Among 9 subjects with past TB, 7 had completed therapy a mean of 3 years prior to sample collection (range=<1 year to 5 years); one subjects had completed therapy in Viet Nam at least 1 year before; and the date of treatment was unknown for 1 subject. Seven had been treated for pulmonary TB; one had treated genitourinary TB, and one had been treated for pleural disease.

### Serologic

**Figure 1:**
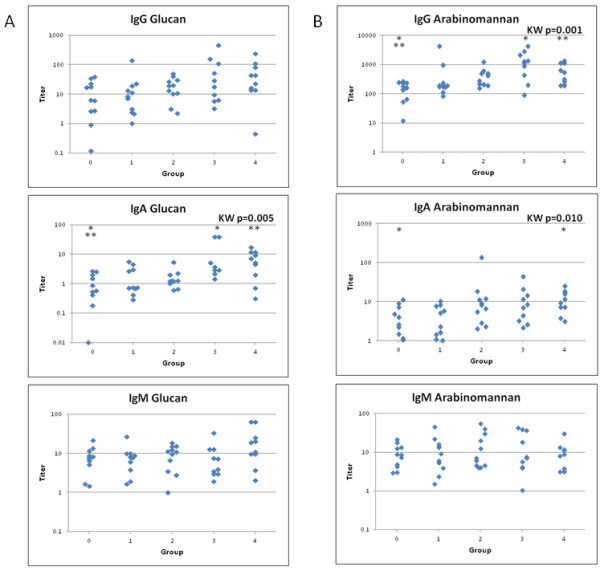
**Panel A shows antibody titers against glucan and panel B shows antibody titers against arabinomannan.** KW is Kruskal-Wallis and significant P values are indicated for across group comparisons. Significant results for Dunn’s post-hoc analysis between groups are indicated by asterisks (within a test, groups with the same number of asterisks are significantly [p<0.05] different from each other). Group 0 is not infected and not exposed; group 1 is exposed, not infected; group 2 is latent tuberculosis; group 3 is active tuberculosis; and group 4 is previously documented active tuberculosis without evidence of current disease.

Antibodies to both polysaccharides were detectable in all groups (Table 2 and Figure 1). There were no differences in IgM antibody levels among the five groups for either polysaccharide (p>0.25 for both AM and Glu; KW test). There were, however, significant differences between the groups for IgA and IgG levels to AM (p=0.010 and 0.001, respectively, KW test) and for IgA antibody levels to Glu (p=0.005). Median IgG levels against Glu were not significantly different between the five groups (p=0.08, KW test). Although there was an overlap in the distribution of antibody levels between all groups, the median IgA antibody levels to AM and Glu and the median IgG antibody levels to AM in subjects with previously documented active TB were higher than unexposed subjects with a negative PPD (p≤0.05 for all comparisons, Dunn’s post hoc test). Similarly, for subjects with active TB, the median levels of IgG anti AM test and IgA anti Glu were higher than levels in unexposed subjects with a negative PPD (group 0; p<0.05; Dunn’s pos hoc test). Median IgG anti-Glu levels in Groups latent, active TB or previously documented active TB were higher than in unexposed PPD-negative subjects, but the differences were not significant. Of note, the median IgG anti-Glu of subjects with previously documented active TB (43.4) was 7 times higher than that of unexposed subjects who were PPD-negative (6.3).

**Table 2:** **Median serum antibody levels (range) against capsular polysaccharides of *Mycobacterium tuberculosis *(arabinomannan and glucan) according to TB class**

**TB Class**	**Arabinomannan (μg/mL)**			**Glucan (μg/mL)**		
	**IgM**	**IgA**^**a**^	**IgG**^**a**^	**IgM**	**IgA**^**a**^	**IgG**
0	8.6	3.3	163	7.9	0.7	6.3
	(2.98–21.7)	(1.05–11.1)	(11.5–262)	(1.4–21.0)	(0.01–2.6)	(0.12–38.5)
1	9.2	2.2	184	7.8	0.7	8.3
	(1.5–45.7)	(1.0–10.1)	(81.4–4262)	(1.6–26.7)	(0.3–5.6)	(1.0–137)
2	7.1	8.2	271	10.9	1.2	19.2
	(4.1–55.7)	(2.0–135)	(154–1222)	(1.0–18.4)	(0.6–5.3)	(2.2–48.4)
3	7.4	7.7	1181^b^	5.5	3.3^b^	23.3
	(1.0–42.3)	(2.2–42.9)	(89.3–4227)	(1.9–32.7)	(1.5–38.5)	(3.2–464)
4	8.9	11.5^b^	524^b^	10.7	5.2^b^	43.4
	(3.1–30.5)	(3.1–24.6)	(189–1301)	(2.0–62.3)	(0.3–16.9)	(0.4–240)

^a^ p≤0.01, Kruskal–Wallis test (with group 0 as control group).

^b^ p<0.05; Dunn’s post–hoc test adjusted for multiple comparisons.

## Discussion

Serum antibodies of the three major isotypes to two CPs of Mtb in HIV-negative adults stratified according to their tuberculosis status were measured by ELISA with purified antigens. Antibodies to both polysaccharides were detectable in all groups. Importantly, the Ig A anti-Glu levels were significantly higher in the active and treated TB groups compared to unexposed subjects who were PPD-negative. Antibodies against AM demonstrated a similar pattern, with the exception that IgG anti-AM was higher in subjects with active TB or previously documented active TB, and the IgA anti-AM was higher in subjects with previously documented active TB when compared with unexposed, PPD-negative subjects. Serum IgG anti-Glu levels were higher in groups with active TB or previously documented active TB than in a group of unexposed, PPD-negative subjects, but the differences were not significant. Thus, with this small number of subjects, groups of patients who had active or treated disease could be identified by the level of antibodies to these two CPs. These data suggest that assays based on antibody responses to the CPs of Mtb may be of value for the serodiagnosis of TB.

What could be the origin of CP antibodies in unexposed, PPD-negative people? Similar to other capsulated pathogens, cross-reactive anti-polysaccharide antibodies were probably generated by exposure to environmental mycobacteria or non-pathogenic enteric or pulmonary bacteria [13-15]. In fact, we have previously shown that the development of low level humoral immune responses to *Mycobacterium avium* sonic extracts and mycobacterial LAM in children correlates with increasing age, and by 18 years of age many individuals were seroreactive to mycobacterial antigens [16]. This could explain the levels observed in the adults with no history or evidence of TB or of exposure to Mtb. The presence of pre-existing anti-CP antibodies could confound use of this method in TB diagnostics in areas with high exposure to environmental mycobacteria or in countries where BCG vaccination is routine.

The role of antibodies in the pathogenesis of tuberculosis and protection against Mtb infections is controversial. Further, the impact of specific antibody isotypes on TB infections is uncertain. The role of serum anti-CPs is under study, but there are accumulating data that this immune component may be protective [4,17-22]. The capsule of Mtb has been shown to limit the association of the organism with macrophages [21]. The antiphagocytic nature of the capsule is similar to that of other encapsulated organisms. Antibody to the CPs of Mtb may therefore enhance the uptake of organisms. A protein-conjugate vaccine containing AM has been shown to reduce the mycobacterial burden in vaccinated mice [4]. Likewise, administration of a monoclonal antibody to the AM moiety of lipoarabinomannan to mice resulted in a dose-dependent reduction in splenic and lung bacterial loads and promoted survival [18]. Interestingly, most of the protective antibodies have an IgG isotype. Our data regarding the serum antibody responses of healthy and Mtb-infected adults to the two Mtb CPs provide information that may be useful in evaluating the effect of CP-based vaccines. Clearly, the pre-existing anti-CP antibody titers should be assessed in volunteers prior to immunization with TB CP vaccines. In initial studies of the safety and immunogenicity of these vaccines, volunteers should have low anti-CP antibody titers to preclude pre-existing antibody interference with the analysis of vaccine activity. Subsequent studies could evaluate whether volunteers with high pre-existing anti-CP antibody levels could be boosted by vaccination.

## Conclusions

In summary, serum antibody levels against surface CPs were shown to be higher among subjects with active or treated tuberculosis. Similar results showing higher AM IgG antibody levels in TB patients than in controls, and higher in TB-HIV negative than in TB-HIV positive patients, were published recently [23]. Our data provide quantitative evidence that disease caused by Mtb elicits an immune response to the CPs. The small sample sizes in our study may have interfered with the ability to detect significant differences among groups for several isotypes of antibody. Expanded studies of defined populations are indicated in order to assess the utility of these assays for epidemiologic and/or clinical purposes.

## Competing interests

None of the living authors have competing interests; no competing interests are known for RWA.

## Authors’ contributions

WAK: Study design; data acquisition, analyses and interpretation; manuscript preparation and revision. ZDD: Antibody purification and detection assay; study design; manuscript preparation and revision. RWA: Study design; data acquisition. RLA: Data analyses and interpretation; manuscript preparation and revision. SM: Antigen preparation; manuscript review (Internal funding). RS: Supervision of antibody purification and detection assay; study conception and design; data interpretation; manuscript preparation, revision and approval. JBR: Supervision of antibody purification and detection assay; study conception and design; data interpretation; manuscript pr eparation, revision and approval. All authors (with the exception of Dr. Awe) read and approved the final manuscript.

## Authors’ information

Dr. Rachel Schneerson, Dr. John Robbins and Dr. ZhongDong Dai these authors are retired.

## Pre-publication history

The pre-publication history for this paper can be accessed here:

http://www.biomedcentral.com/1471-2334/13/276/prepub
